# Mechanisms of methotrexate resistance in osteosarcoma cell lines and strategies for overcoming this resistance

**DOI:** 10.3892/ol.2014.2773

**Published:** 2014-12-05

**Authors:** JIANJUN WANG, GUOJUN LI

**Affiliations:** 1 Department of Oncology, Henan University Huaihe Hospital, Kaifeng, Henan 475001, P.R. China; 2 Department of Orthopedics, Henan University Huaihe Hospital, Kaifeng, Henan 475001, P.R. China

**Keywords:** osteosarcoma cell lines, drug resistance, methotrexate, overcoming resistance

## Abstract

The aim of the present study was to investigate the underlying mechanisms of methotrexate (MTX) resistance in the human osteosarcoma cell line, Saos-2/MTX4.4, and to evaluate various methods of overcoming the resistance to this chemotherapeutic agent. MMT assays were performed to determine the resistance of the primary (Saos-2) and resistant (Saos-2/MTX4.4) cell lines to MTX, cisplatin [cis-diamminedichloroplatinum II (DDP)], ifosfamide (IFO), Adriamycin (ADM), epirubicin (EPI) and theprubicin (THP). The Saos-2/MTX4.4 cells exhibited a low resistance to IFO, ADM, EPI and THP; however, no resistance to DDP was identified. Overall, the Saos-2/MTX4.4 cells exhibited a greater resistance to all the chemotherapeutic agents investigated compared with the Saos-2 cells. Rhodamine 123 (R123) fluorescence was measured in the Saos-2/MTX4.4 and Saos-2 cells 30 and 60 min after the addition of R123, and R123 plus verapamil (VER). VER administration increased the intracellular accumulation of R123. In addition, reverse transcription-quantitative polymerase chain reaction was performed to determine the mRNA expression levels of multidrug resistance gene 1 (MDR1) in the two cell lines. Although the Saos-2/MTX4.4 cells were more resistant to the chemotherapeutic agents than the Saos-2 cells, no significant difference was identified between the relative mRNA expression levels of MDR1 in the Saos-2/MTX4.4 and Saos-2 cells (0.4350±0.0354 vs. 0.3886±0.0456; P>0.05).

## Introduction

Osteosarcoma is a common type of bone cancer that predominantly occurs during adolescence, and is clinically characterized by local infiltration and early, distant, hematogenous metastasis (1–3). Although the prognosis of osteosarcoma patients has significantly improved since the implementation of a comprehensive treatment strategy using surgery and adjuvant chemotherapy (4–7), the prognosis remains poor for a number of patients due to the development of acquired resistance. Intrinsic resistance is a phenomenon that occurs prior to chemotherapy and is not associated with the administration of chemotherapeutic agents, whereas acquired resistance is induced by chemotherapeutic agents (8,9). In clinical practice, adjuvant chemotherapy for osteosarcoma generally includes the administration of methotrexate (MTX), cisplatin [cis-diamminedichloroplatinum II [DDP)], ifosfamide (IFO), doxorubicin, pirarubicin or a combination of these agents. High-dose MTX is considered to be the key agent in determining the chemotherapeutic outcome of osteosarcoma patients (10); however, multidrug resistance often develops in the late stage of treatment. In the present study, shock treatment and a gradually increasing dose of MTX were used to investigate acquired resistance in the MTX-resistant osteosarcoma cell line, Saos-2/MTX4.4. Our previous study demonstrated that MTX induces resistance in MTX-resistant cell lines (11). This resistance may be associated with the downregulation of folate carrier gene expression levels, as well as the reduced cellular influx of MTX, reducing the ability of MTX to competitively inhibit tumor cell DNA synthesis (12–15). In the present study, the multidrug resistance of the Saos-2/MTX4.4 cell line was investigated. Furthermore, the association between multidrug resistance and multidrug resistance gene 1 (MDR1) overexpression was determined in the presence of substrates of P-glycoprotein (Pgp), a product of the MDR1 gene. The aim of the present study was to investigate the efficacy of verapamil (VER) in preventing Pgp pumping MTX out of the cell, in order to overcome MTX resistance in osteosarcoma treatment.

## Materials and methods

### Cell culture and MTT assay

Human osteosarcoma Saos-2 cells (Shanghai Institutes for Biological Sciences, Chinese Academy of Sciences, Shanghai, China) were exposed to shock therapy using gradually increasing concentrations of MTX (1.1, 2.2 and 4.4 μM; Shanghai New Hualian Pharmaceutical Co., Ltd., Shanghai, China) to create an MTX-resistant cell line. The Saos-2 parent cells were cultured in RPMI-1640 medium (Invitrogen Life Technologies, Carlsbad, CA, USA) with 10% fetal bovine serum (HyClone, Logan, UT, USA) ) and 0.2% penicillin/streptomycin (Sigma-Aldrich, St. Louis, MO, USA), at 37°C and 5% CO_2_. When the cells had reached 60–70% confluence in the logarithmic growth phase, 4.4 μM MTX was added. After 24 h, the cells were washed twice with 1X phosphate-buffered saline (PBS) at 37°C, and an agent-free medium was added to the cells. Once the cells had grown to 60–70% confluence in a logarithmic phase, the process was repeated three times at each MTX concentration. Following seven months of resistance-induction, the MTX-resistant cell line, termed Saos-2/MTX4.4, was established and compared with the primary Saos-2 cells.

An MTT assay (Sigma-Aldrich) was used to determine the sensitivity of the Saos-2 and Saos-2/MTX4.4 cells to MTX, IFO (Jiangsu Henrui Medicine Co., Ltd., Jiangsu, China), DDP (Haosen Pharmaceutical Co., Ltd., Jiangsu, China), Adriamycin (ADM; Zhejiang Haizheng Pharmaceuticals, Taizhou, China), epirubicin (EPI; Pfizer, Inc., Madison, NJ, USA) and theprubicin (THP; Shenzhen Wanle Pharmaceutical Co., Ltd., Shenzhen, China). Logarithmic growth phase cells were suspended in RPMI-1640 medium, seeded in a 96-well plate at 200 μl/well (1×10^4^ cells) and cultured for 24 h. Based on the peak plasma concentrations of the clinical agents, seven concentration gradients (1000, 100, 10, 1, 0.1, 0.01 and 0.001 plasma protein concentration) of MTX, DDP, IFO, EPI and THP were added. Untreated cells were used as the control. After 24 h of incubation, 20 μl of 5% MTT was added to each well, and after another 4 h of incubation, the culture supernatant was removed, 150 μl dimethylsulfoxide was added to each well and the cell mixture was agitated for 10 min at room temperature. A Wellscan MK3 microplate reader (Labsystems Dragon, Helsinki, Finland) measured the light absorption of the cells at a wavelength of 490 nm. The inhibitory rate of the cells was determined using the following formula: Inhibitory rate = (1 - mean absorption value of resistance group/mean absorption value of control group) × 100. The median inhibitory concentration (IC_50_) was identified and the resistance index (RI) of the two cell lines was determined using the following formula: RI = IC_50_(Saos-2/MTX4.4)/IC_50_(Saos-2).

### Rhodamine 123 (R123) efflux

The Pgp activity of the cell lines was determined by evaluating R123 efflux. R123 is a Pgp-specific fluorescent substrate and its intracellular accumulation is associated with the efficiency of Pgp activity. The changing rate of R123 efflux was determined using the following equation: Rate of efflux = (fluorescence intensity of experimental group - fluorescence intensity of control group)/fluorescence intensity of control group × 100. Equal quantities of Saos-2 and Saos-2/MTX4.4 cells were seeded on a 96-well plate (density, ~1×10^5^ cells/well) and cultured in RPMI-1640 medium for 24 h. The cells were divided into four groups: Primary cells (Saos-2; group A), MTX-resistant cells (Saos-2/MTX4.4; group B), primary cells treated with VER (group C) and MTX-resistant cells treated with VER (group D). VER was added to the wells containing group C and D cells at a final concentration of 4.5 μM 1 h prior to the addition of R123. R123 was added into all wells at a final concentration of 0.15 μg/ml. Each group was replicated in six wells and incubated in an atmosphere of 5% CO_2_ and 100% humidity. Three wells from groups C and D were harvested 30 min after incubation with VER and R123, and the other three wells were harvested 60 min after incubation. Subsequent to being harvested, these cells were rinsed twice with PBS, resuspended in RPMI-1640 medium and observed using fluorescence microscopy (IX17; Olympus Corporation, Tokyo, Japan). Following a further 4 h of incubation, all the cells were harvested, washed twice with D-Hank’s solution (Shanghai Jiaotong University Laboratory, Shanghai, China) and digested at 4°C overnight in a 50% ethanol solution containing 0.3 mol/l HCl. The fluorescence intensity was measured using a multifunctional microplate reader (Wellscan MK3) at an excitation wavelength of 485 nm and an emission wavelength of 538 nm.

### Semiquantitative analysis of MDR1 mRNA expression

Total RNA was extracted from the cells using TRIzol reagent (Invitrogen Life Technologies) and was subjected to reverse transcription-quantitative polymerase chain reaction (RT-qPCR) using a PrimeScript RT reagent kit (Takara Bio, Inc., Shiga, Japan), according to the manufacturer’s instructions. Gene-specific primer pairs were designed based on the human MDR1 and β-actin complementary DNA sequences (GenBank Assembly ID: GCA_000161855.1) from Genbank (www.ncbi.nlm.nih.gov/genbank) and were synthesized by Sangon Biotech Co., Ltd (Shanghai, China). The primer sequences were as follows: Forward, 5′-GTT GCT GCT TAC ATT CAG GTT TC-3′ and 5′-ACC AGC CTA TCT CCT GTC GC-3′ for MDR1; and forward, 5′-AAC TGG GAC GAC ATG GAG AAA ATC-3′ and reverse, 5′-AGG AAG GAA GGC TGG AAG AGT GC-3′ for β-actin. PCR reactions were performed using GeneAmp PCR System 9700 (Applied Biosystems Life Technologies, Foster City, CA, USA), with pre-denaturation at 95°C for 5 min, followed by 35 cycles at 94°C for 30 sec, 64°C for 30 sec and 72°C for 60 sec.

### Statistical analysis

All experiments were conducted in triplicate. Data were analyzed using SPSS software (version 13.0; SPSS, Inc., Chicago, IL, USA), and are expressed as the mean ± standard deviation. Comparisons between two groups were performed using one-way analysis of variance, and P<0.05 was considered to indicate a statistically significant difference.

## Results

### MTT assay

The IC_50_ of the Saos-2/MTX4.4 cells to MTX was 12.73 times higher than that of the Saos-2 cells, giving an RI of 12.73, indicating that Saos-2/MTX4.4 cells exhibit moderate resistance to MTX. The Saos-2/MTX4.4 cells demonstrated lower resistance to IFO, ADM, EPI and THP; however, the Saos-2/MTX4.4 cells exhibited no evident cross-resistance to DDP (Table I).

### R123 efflux in the Saos-2/MTX4.4 and Saos-2 cells

**Figure 1:**
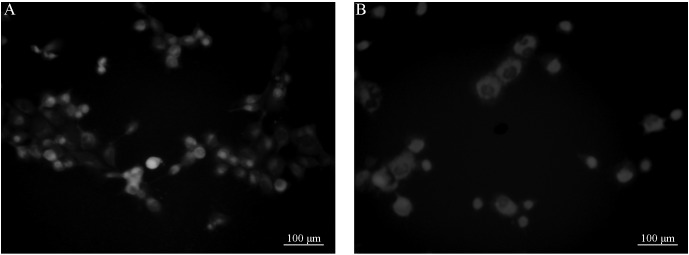
Fluorescence intensity of group A cells: Saos-2 cells at (A) 30 and (B) 60 min after rhodamine 123 administration.

**Figure 2:**
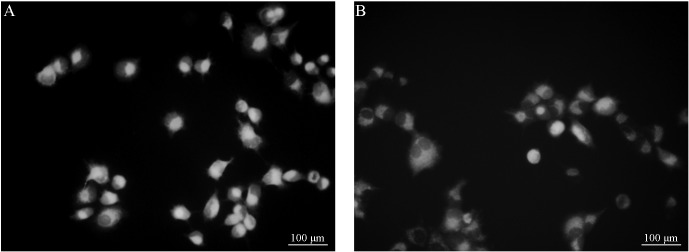
Fluorescence intensity of group B cells: Saos-2/MTX4.4 cells at (A) 30 and (B) 60 min after rhodamine 123 administration.

**Figure 3:**
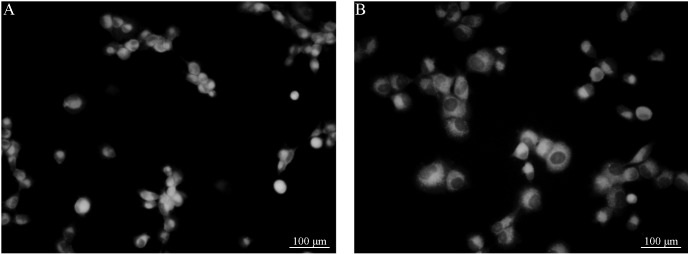
Fluorescence intensity of group C cells: Saos-2 cells at (A) 30 and (B) 60 min after rhodamine 123 and verapamil administration.

**Figure 4:**
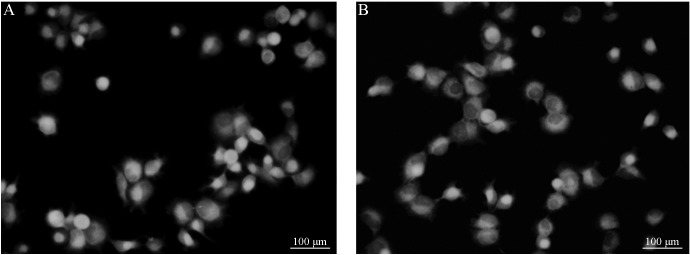
Fluorescence intensity of group D cells: Saos-2/MTX4.4 cells at (A) 30 and (B) 60 min after rhodamine 123 and verapamil administration.

Fluorescence microscopy demonstrated that 30 or 60 min post-incubation, the cells in groups A and B exhibited full shapes or multiple pseudopodia. In addition, 30 or 60 min post-incubation, the cells in groups C and D also exhibited full shapes or multiple pseudopodia. However, no significant differences in cell morphology were observed between the groups. Furthermore, the intracellular fluorescence intensity of the cells was apparent, and no significant differences were identified between groups A and B (Figs. 1 and 2). Following treatment with VER, the fluorescence intensity of the group C cells demonstrated no significant difference from that of the group D cells (Figs. 3 and 4). However, the cells in groups C and D demonstrated a marked difference in fluorescence intensity compared with cells from groups A and group B, respectively. Considering that the cells were digested and detected using a multifunctional microplate reader, the results are consistent with the observations obtained from fluorescence microscopy. No significant difference was identified between the R123 fluorescence intensity of the primary and resistant cells. However, significant differences were observed prior to and following the addition of VER in the primary and resistant cell groups (P<0.05; Table II).

### MDR1 mRNA expression in Saos-2/MTX4.4 cells

The MDR1 mRNA expression level is expressed as the ratio of the optical density of MDR1 RT-qPCR products to the optical density of the β-actin RT-qPCR products. The optical density of the β-actin band in each lane was set to one and the relative MDR1 mRNA expression was calculated using gel-imaging system analysis software (Quantity One v4.6; Bio-Rad Laboratories, Hercules, CA, USA). The MDR1 mRNA relative expression levels in the Saos-2/MTX4.4 and Saos-2 cells were 0.4350±0.0354 and 0.3886±0.0456, respectively. A t-test identified no significant difference between the MDR1 mRNA expression levels in the Saos-2 and Saos-2/MTX4.4 cells.

## Discussion

The concept of the multidrug resistance of tumors was initially proposed in 1970 (16) and stated that tumor cells exhibit a cross-resistance to a variety of chemotherapeutic agents with different structures and functions. Various basic and clinical studies have indicated that, in the majority of cancers, multidrug resistance is associated with the expression of the MDR1 gene. The protein product of MDR1 is P-170, a membrane glycoprotein that functions as an energy-dependent drug pump. P-170 actively exports various anticancer drugs and hydrophobic compounds to reduce the intracellular drug concentration, ultimately resulting in drug resistance (17–24). Numerous studies (17–24) have indicated that doxorubicin-resistant cell lines generally exhibit increased MDR1 gene expression. In the present study, the induced MTX-resistant cell line also showed multidrug resistance. Schwartz *et al* (19) used an immunohistochemical assay to investigate Pgp expression in biopsy specimens from 685 osteosarcoma patients prior to treatment with chemotherapy, and identified that Pgp expression was not correlated with patient prognosis. However, Baldini *et al* (25) supported the finding that the Pgp expression level predicts the clinical effects of doxorubicin-based chemotherapy. Thus, Pgp cannot be used as a single predictor of doxorubicin-resistance in the treatment of osteosarcoma. The results of the present study are consistent with these findings. Although the MTX-resistant cell line exhibited multidrug resistance, no significant difference was identified in the expression of the MDR1 gene between the primary and MTX-resistant cells. Furthermore, similar results were identified for R123 efflux; no significant difference was identified in the rate of R123 efflux between the primary and MTX-resistant cells. Thus, MDR1 gene expression may not be the primary cause of multidrug resistance and the efflux of therapeutic agents via the Pgp pathway may not be the primary route of multidrug resistance.

Previously, VER has been reported to reverse multidrug resistance (26,27). Consistent with this, the present study demonstrated that VER significantly increased the efflux of R123, indicating that VER increases the quantity of soluble intercellular agents by inhibiting the function of Pgp, thus increasing the efficiency of chemotherapeutic agents. However, patients are unable to tolerate treatment with VER due to its toxic cardiovascular side-effects, thus limiting the application of VER in clinical practice (28–31). In conclusion, further investigation into the underlying mechanism of VER activity may provide alternative means for the development of novel therapeutics.

## Figures and Tables

**Table I:** Resistance of Saos-2 and Saos-2/MTX4.4 cells to various chemotherapeutic agents [IC_50_ (μmol/l) mean ± standard deviation].

Chemotherapeutic agent	Saos-2	Saos-2/MTX4.4	RI
MTX	25.78±0.29	328.24±0.29	12.73
IFO	583.23±0.14	2346.52±0.37	4.02
DDP	127.67±0.21	156.56±0.87	1.23
ADM	8.80±0.45	34.67±0.23	3.94
EPI	10.68±0.35	43.67±0.46	4.09
THP	12.29±0.72	38.72±0.25	3.15

MTX, methotrexate; RI, resistance index; IFO, ifosfamide; DDP, cisplatin; ADM, Adriamycin; EPI, epirubicin; THP, theprubicin.

**Table II:** Rhodamine 123 fluorescence intensity in four groups at various time-points [(arb. unit) mean ± standard deviation].

	Incubation period, min	
	
Group	30	60
A	4.35±0.20	3.25±0.12
B	4.89±0.32	3.20±0.21
C	6.58±0.45	5.20±0.23
D	5.93±0.23	5.12±0.14^a^

Group A, primary cells (Saos-2); Group B, methotrexate-resistant cells (Saos-2/MTX4.4); Group C, primary cells with verapamil; Group D, methotrexate-resistant cells with verapamil.

a P<0.05 vs. group B.
